# P-1302. Klebsiella pneumoniae Phage Susceptibility Testing (PST): Comparison Between a Spot Plaque Assay and an Optical Density-Based Liquid Assay

**DOI:** 10.1093/ofid/ofaf695.1490

**Published:** 2026-01-11

**Authors:** Luz Cuello, Robin Patel

**Affiliations:** Mayo Clinic Graduate School of Biomedical Sciences, Rochester, MN; Mayo Clinic, Rochester, MN

## Abstract

**Background:**

Phage therapy has gained increasing interest as an approach to treat antimicrobial- resistant bacterial infections. Standardized, reproducible PST methods are needed to rapidly identify active phages for potential clinical use. Here, PST of 24 clinical isolates of carbapenem-resistant *K. pneumoniae* (CR-*Kp*) to 6 *K. pneumoniae* phages (3 from KlebPhaCol and 3 from phage hunting) was assessed using a plaque assay (PA) and a liquid assay (LA), and results compared.

Phage Susceptibility Testing: Agreement Between Plaque Assay and Liquid Assay

**Figure:**
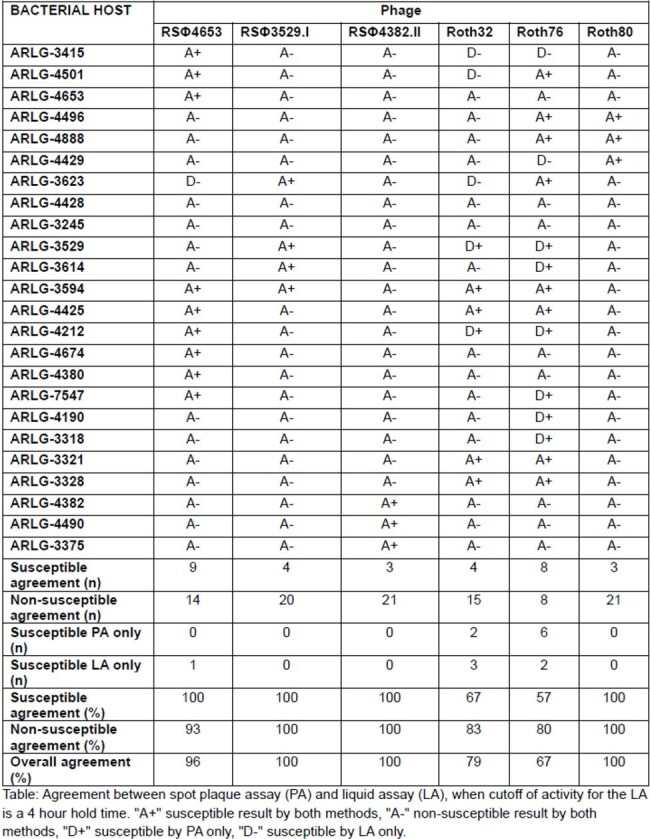

**Methods:**

For the PA, 2µL of 10^−1^-10^−8^ phage dilutions were spotted on bacterial laws of CR-*Kp* (double agar overlay), and plaques scored upon overnight incubation at 37°C [0 (no activity), 1+ (lysis from without), 2+ (concentration-dependent lysis), 3+ (clear plaques, small or medium size), 4+ (clear plaques of large size); scores ≥ 2+ were considered susceptible]. Testing was performed on 3 different days. If ≥ 2 replicates scored ≥ 2+, the result was classified as susceptible.

For the LA, phages and bacteria were combined (MOI=10) in 96-well plates [phage-bacteria combinations (3 replicates), phage controls (10^6^ PFU/mL), bacterial controls (BC, 10^5^ CFU/mL), media sterility controls] and plates incubated at 37°C for 24h, with OD_600_ measured every 15 min. Hold time (HT) — delay in achieving inflection at the beginning of exponential growth phase — was calculated for phage-treated compared to BC wells. Testing was performed on 2 different days. Mean HT was computed for each phage-bacteria combination and results assessed as susceptible or not using cutoff HTs of 4h and 8h.

**Results:**

Using a LA HT cutoff of 4h, there was complete agreement with results of the PA for 3 phages, and 96, 79 and 67% overall agreement for 3 other phages (Table). Using a LA HT cutoff of 8h, there was complete negative agreement for 4 phages, but lower overall agreement between the LA and PA (58-96%). Increased classification as susceptible by PA compared to LA was the main source of disagreement when using a LA HT cutoff of 8h.

**Conclusion:**

Here, two PST assays were compared, and greater agreement was found when using a 4h compared to an 8h HT LA cutoff. These results represent a step forward in the standardization of PST methods, bearing in mind that correlation of results with pre-clinical and clinical outcomes is needed.

**Disclosures:**

Robin Patel, MD, a patent on Bordetella pertussis/parapertussis PCR issued, a patent on a device/method for sonication, a patent on PET imaging of bacterial infection: a patent on Bordetella pertussis/parapertussis PCR issued, a patent on a device/method for sonication, a patent on PET imaging of bacterial infection|MicuRx Pharmaceuticals and bioMérieux: Grant/Research Support|PhAST, Day Zero Diagnostics, DEEPULL DIAGNOSTICS, S.L., Nostics, HealthTrackRx, bioMérieux and CARB-X: Advisor/Consultant|Up-to-Date and the Infectious Diseases Board Review Course: Honoraria

